# Supplementary material for: A Novel Registration Method for a Mixed Reality Navigation System Based on a Laser Crosshair Simulator: A Technical Note
Source: Bioengineering (Basel). 2023 Nov 7;10(11):1290. doi: 10.3390/bioengineering10111290 (PMC10669875; doi:10.3390/bioengineering10111290)

Supplementary Material 1

Protocol: Preparation of a 3D Printed Skull Model with Laser Crosshair Projection Using 3D Slicer Software

Summary

This protocol outlines the process of creating a skull model using 3D Slicer software, aiming to simulate a patient's head under the projection of laser localizing lights on a CT scanner gantry. The protocol provides step-by-step instructions for creating a hollow skull model from CT/MRI data, setting up laser crosshair projections, and exporting the model for 3D printing. This protocol enables the generation of a realistic model allowing for various applications such as surgical planning and device testing.

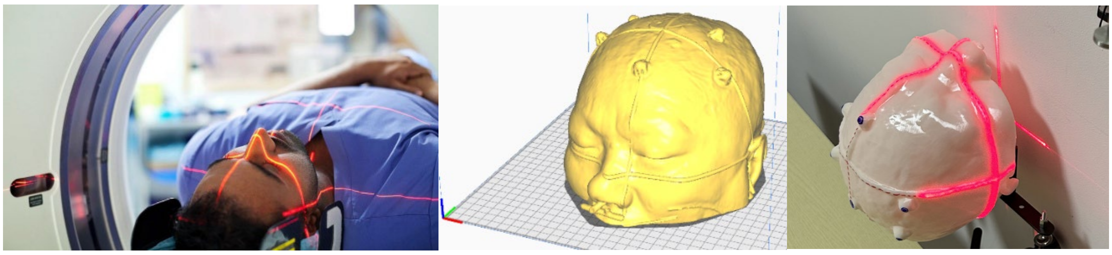

I. Create a Hollow Skull Model

1. Load a CT volume of a head scan.

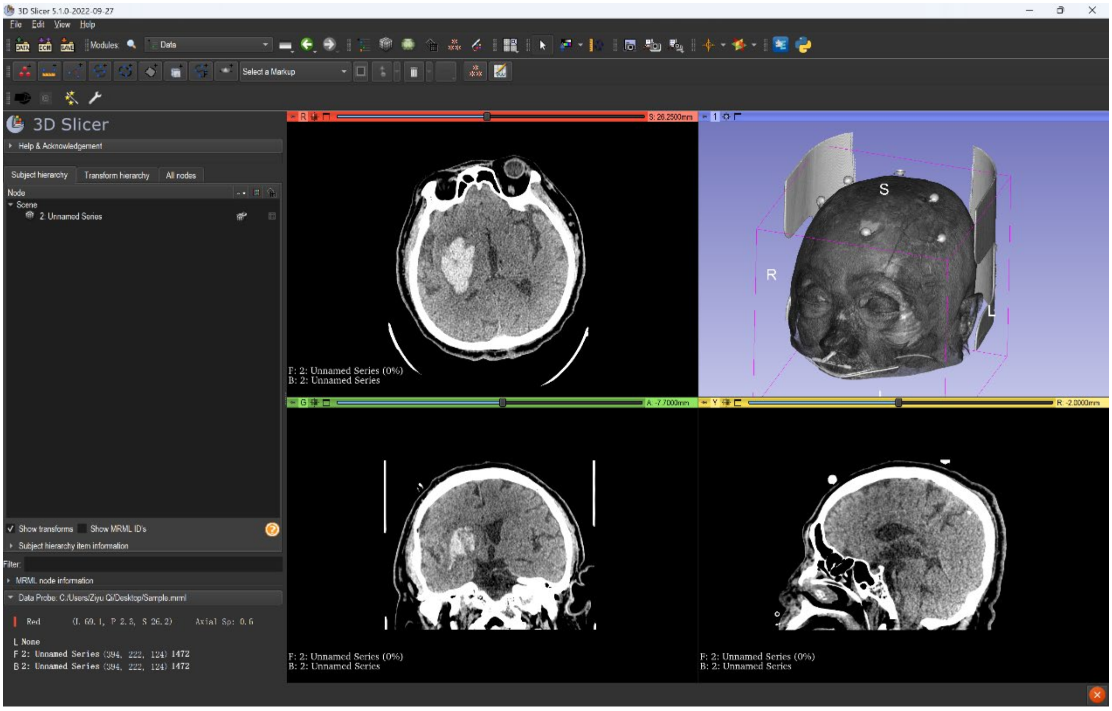

2. Open the Segment Editor module and create a new Segmentation named "Air."

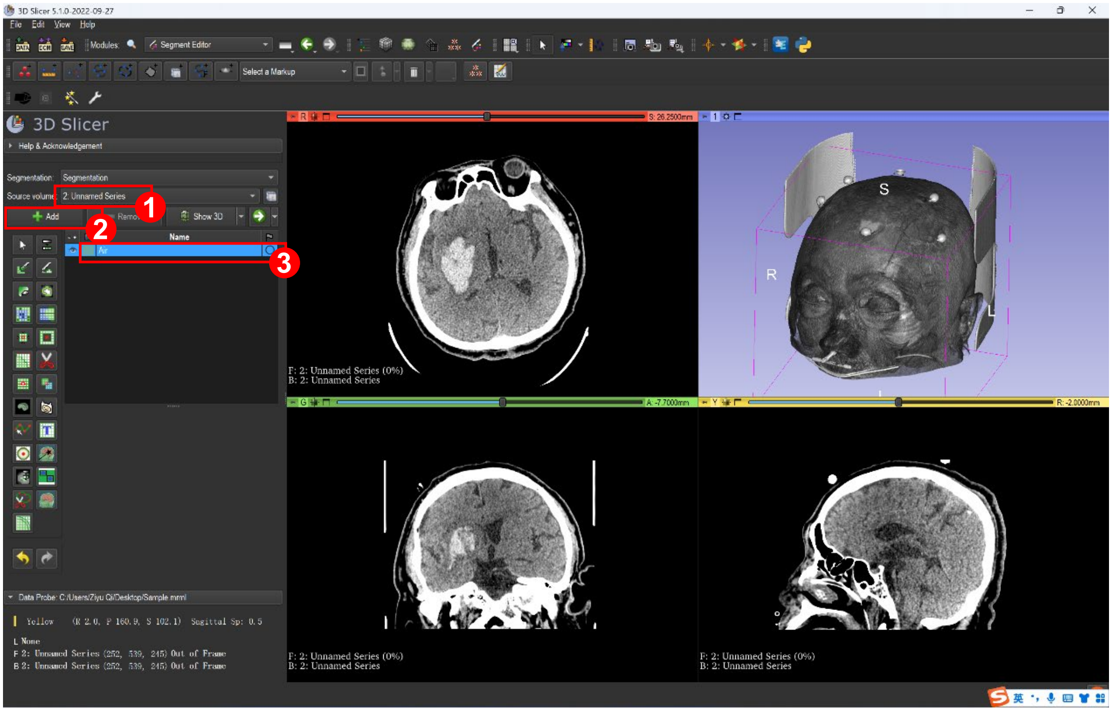

3. Set a threshold range of less than -200 to segment the air-filled regions in the CT volume.

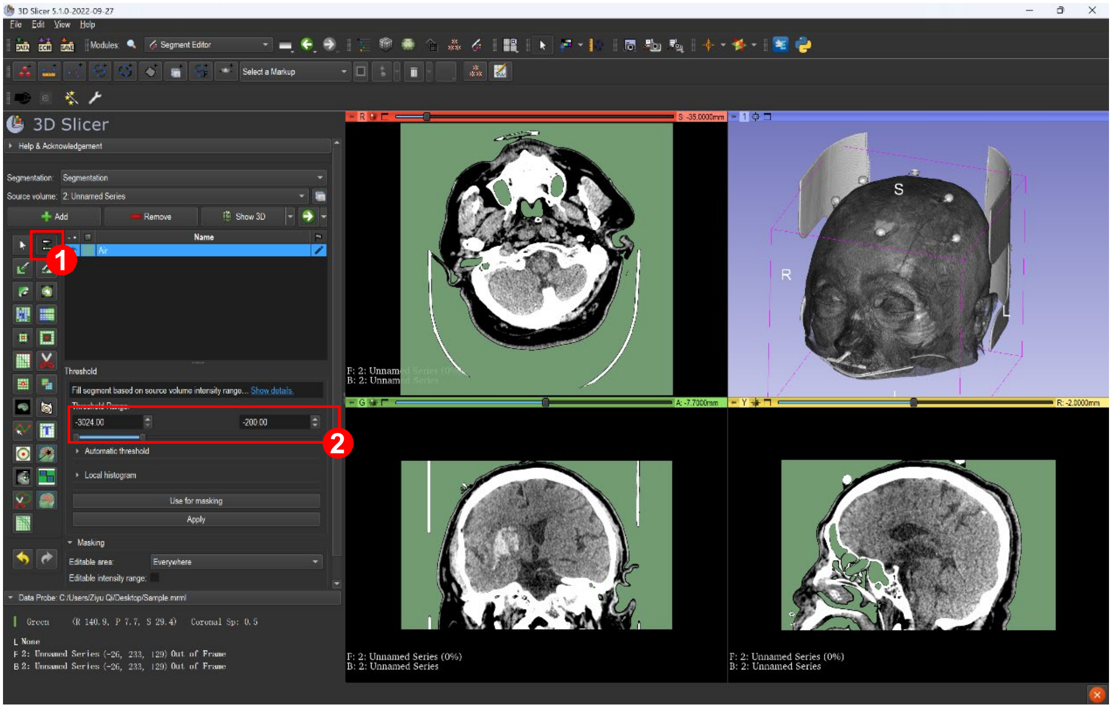

4. Use the Scissors/Erase tool to "cut off" the air connections between the skull and the surrounding air at the skull-neck junction, bilateral external auditory canals, and nasal passages.

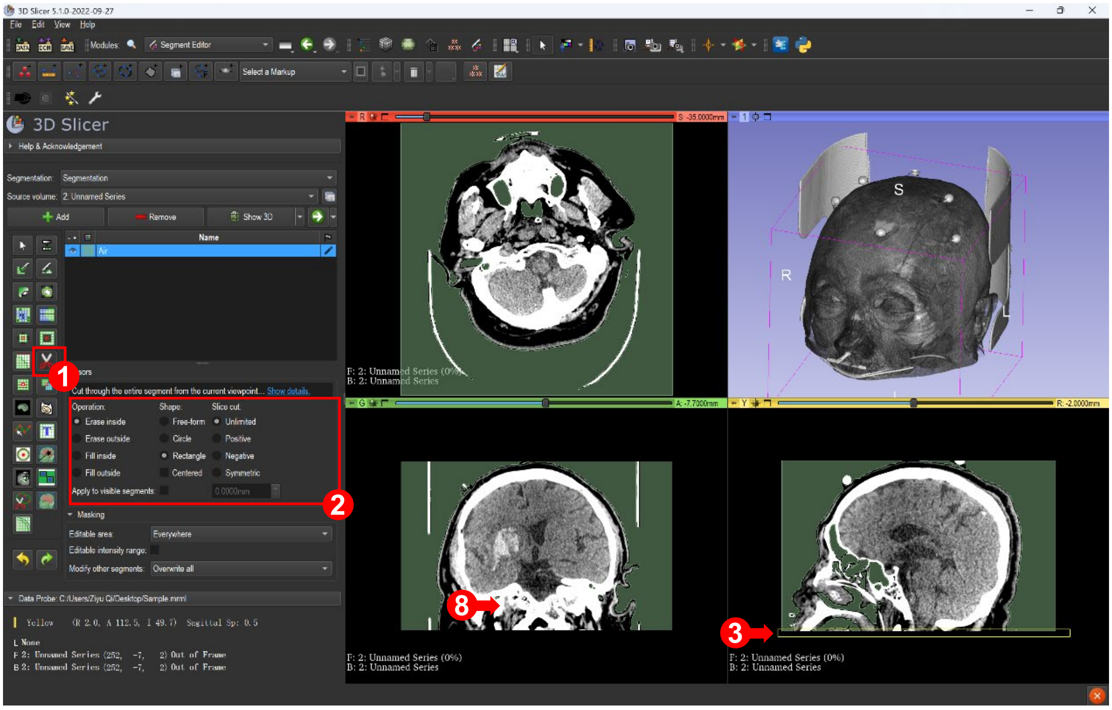

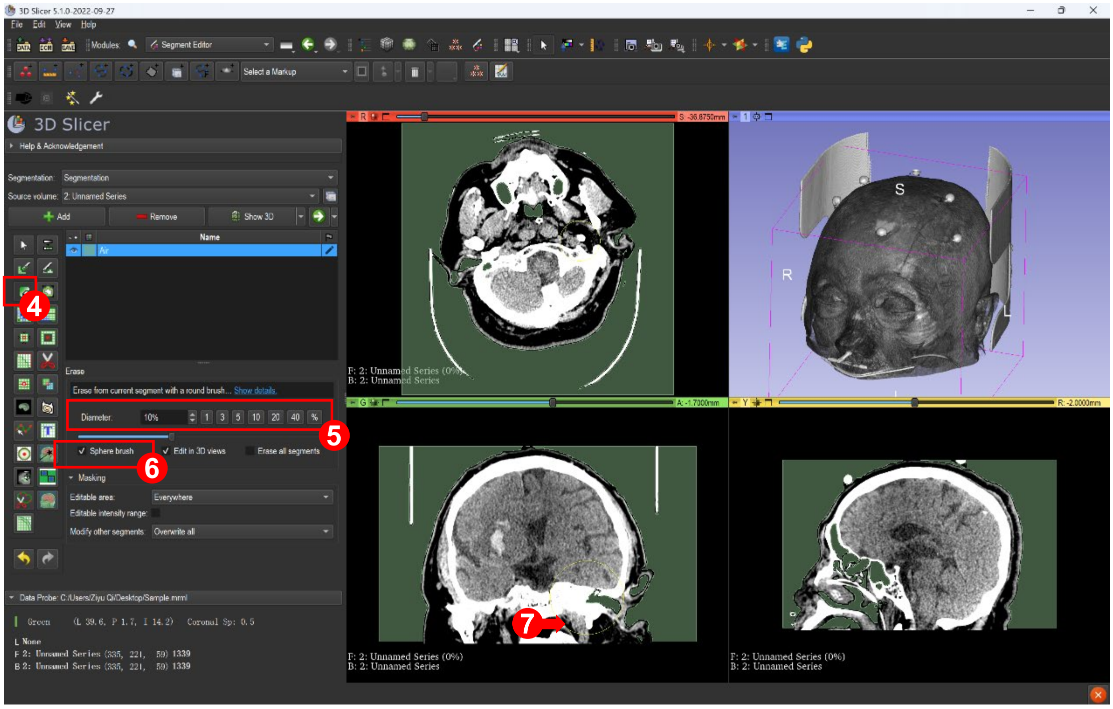

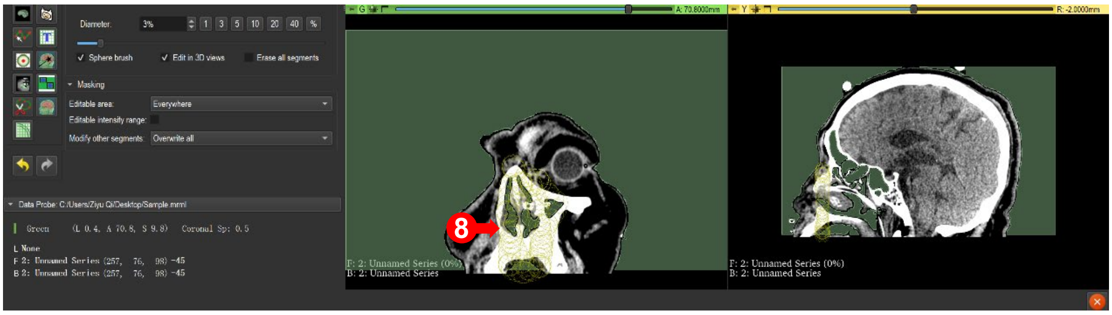

5. Use the Island tool to retain only the gas outside the skull.

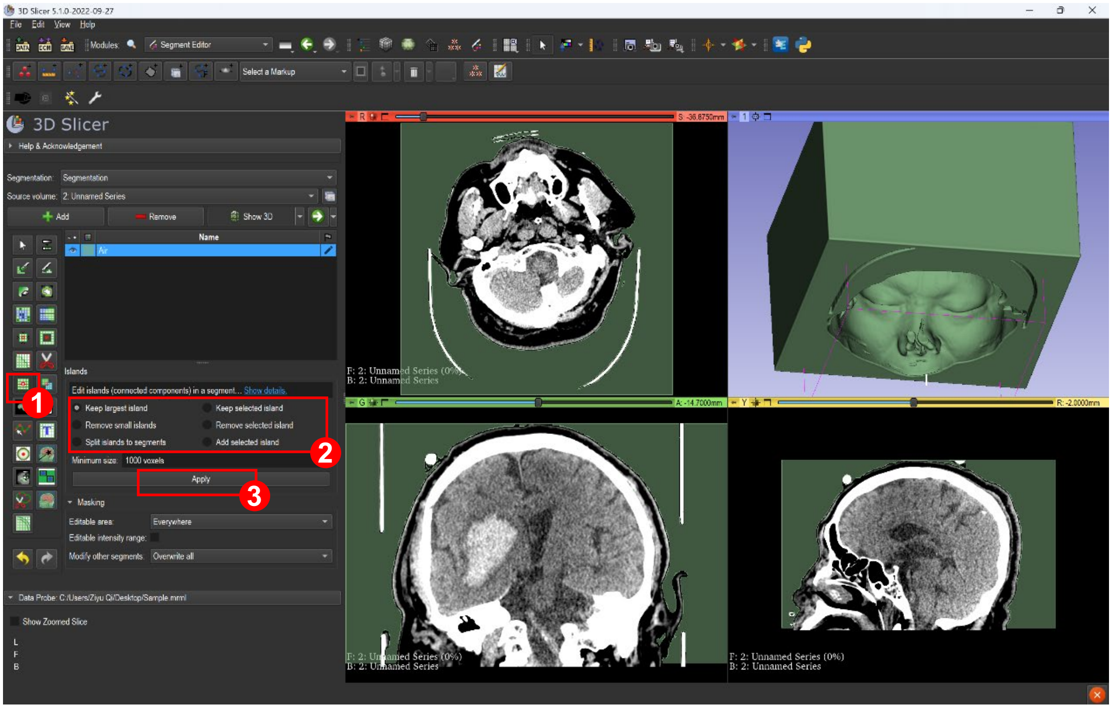

6. Invert the "Air" segment using the Boolean operation tool to obtain the non-gas components (skull and scanning bed structure).

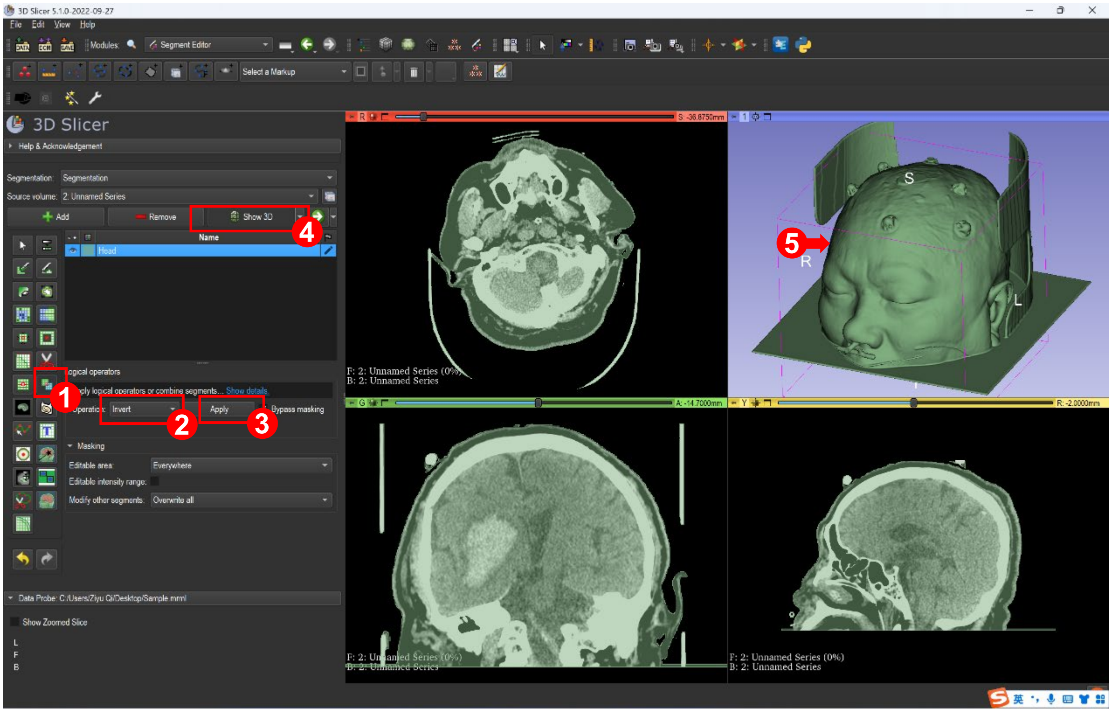

7. Use the Scissors and Island tools to further refine the model, keeping only the skull.

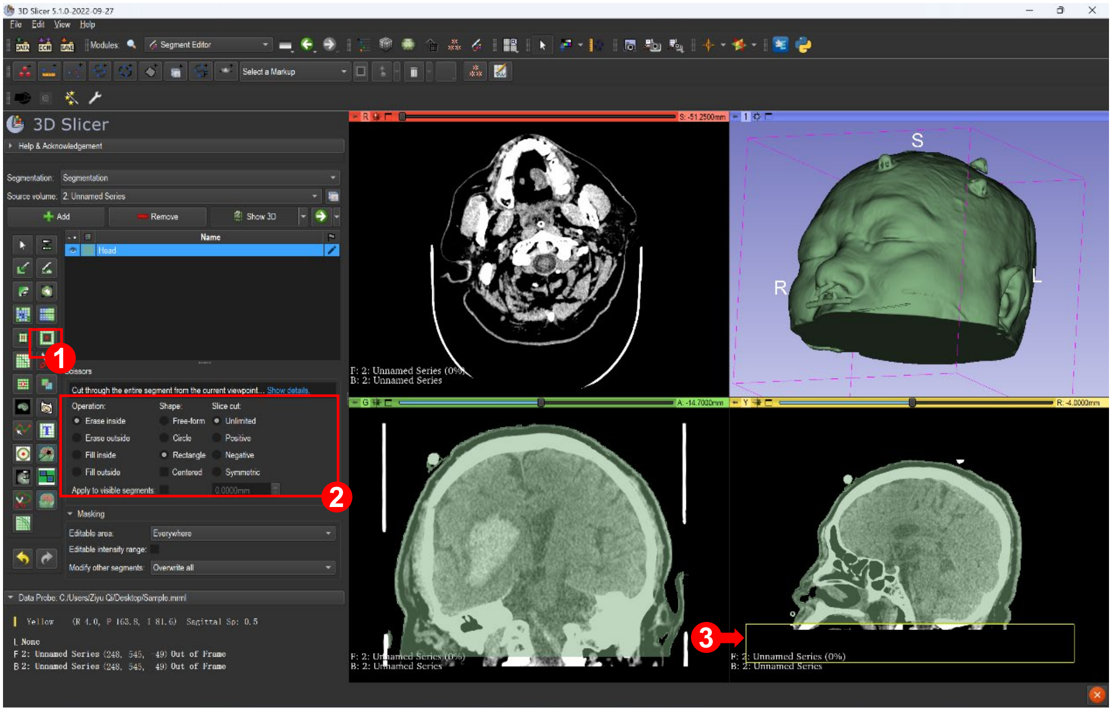

8. Use the "Hollow" tool to create a hollow shell of the skull, removing the internal structures.

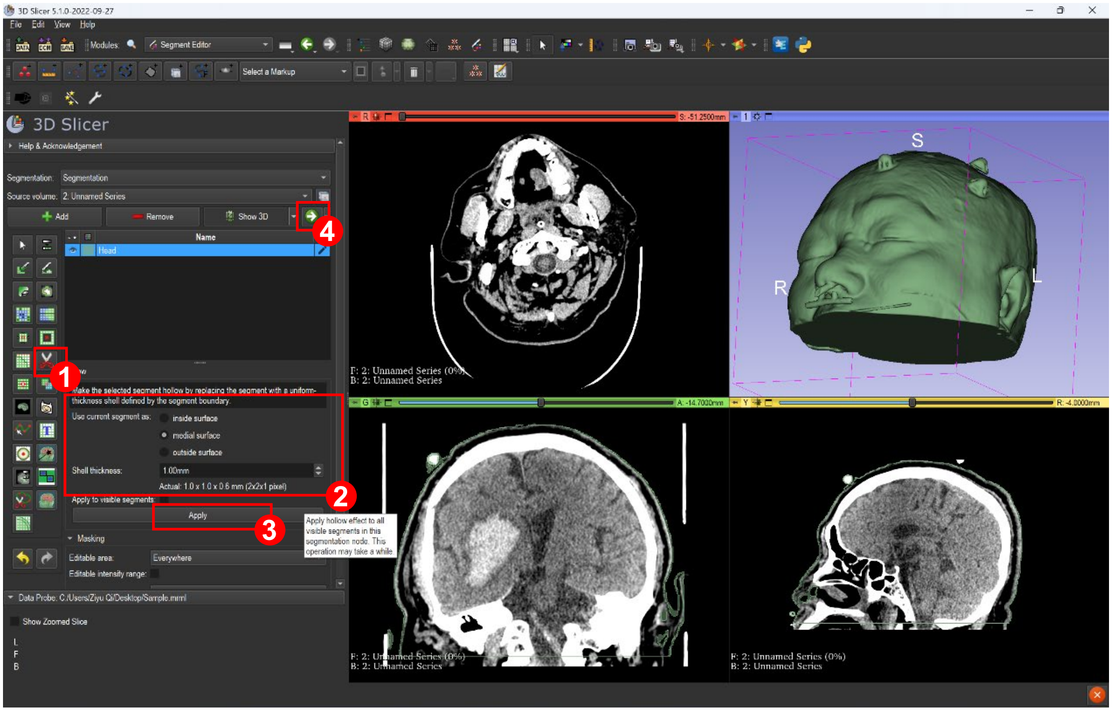

9. Convert the segmentation result into a model.

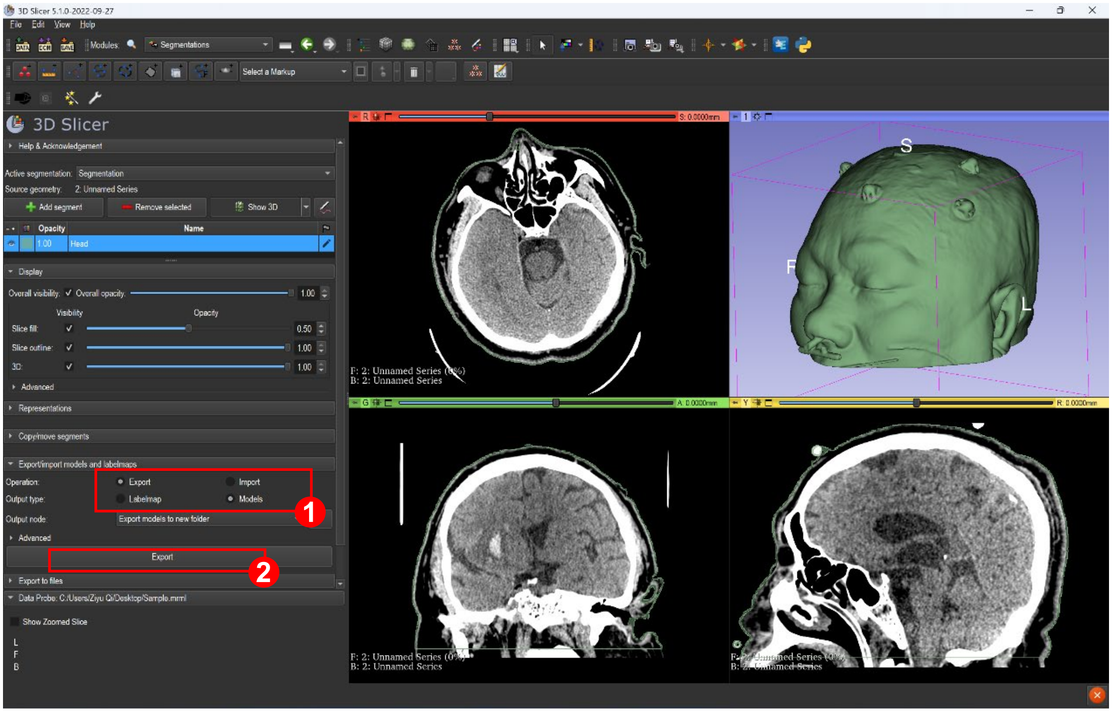

II. Annotation of Laser Crosshair Projection

10. Manually set the sampling interval of the sagittal, coronal, and axial planes to 0.1mm.

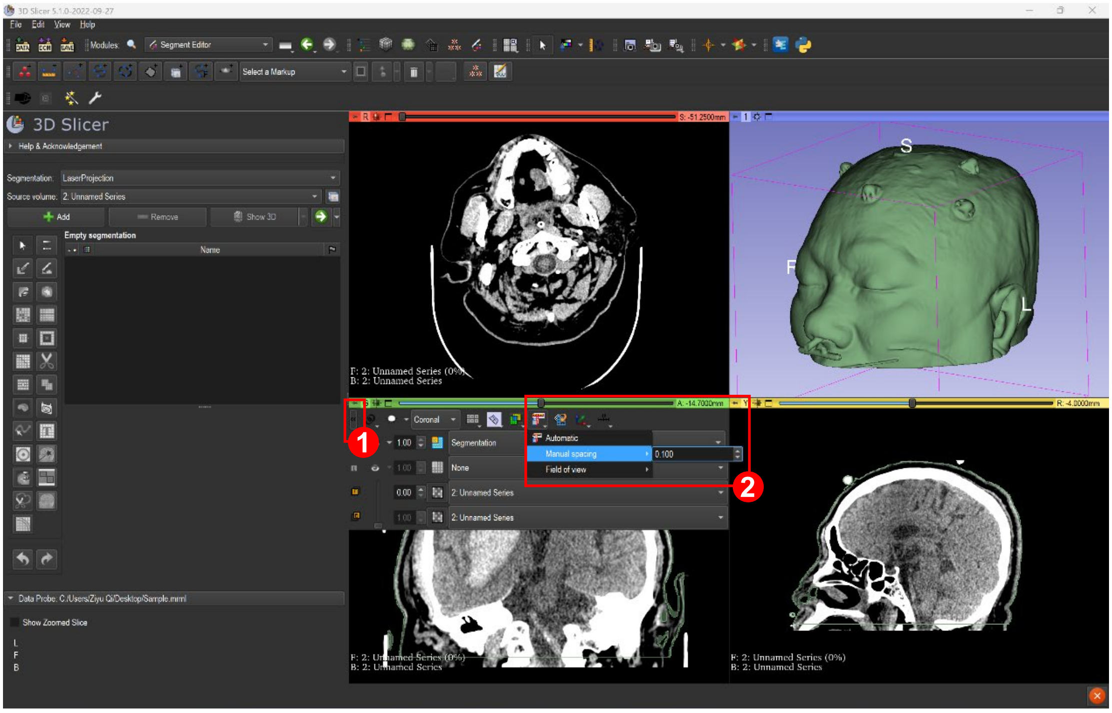

11. Enable the "Basic Crosshair" and adjust the sagittal, coronal, and axial planes individually until the data probe indicates the scanning zero plane. Yellow crosshair markers will appear on each plane.

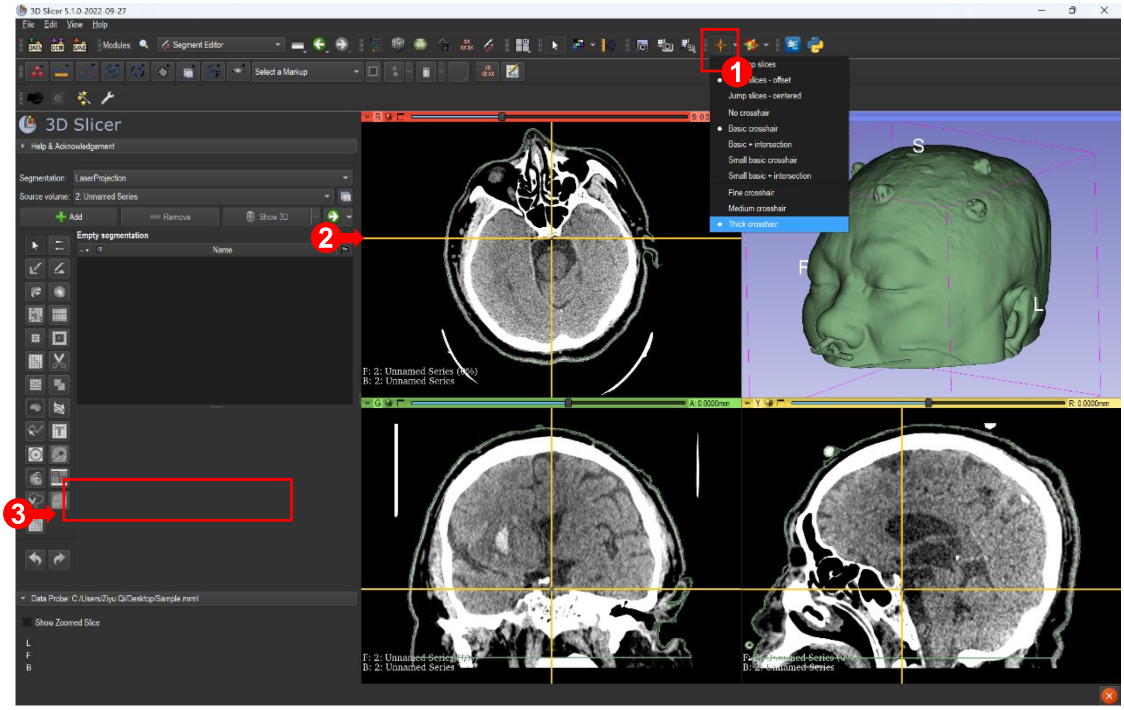

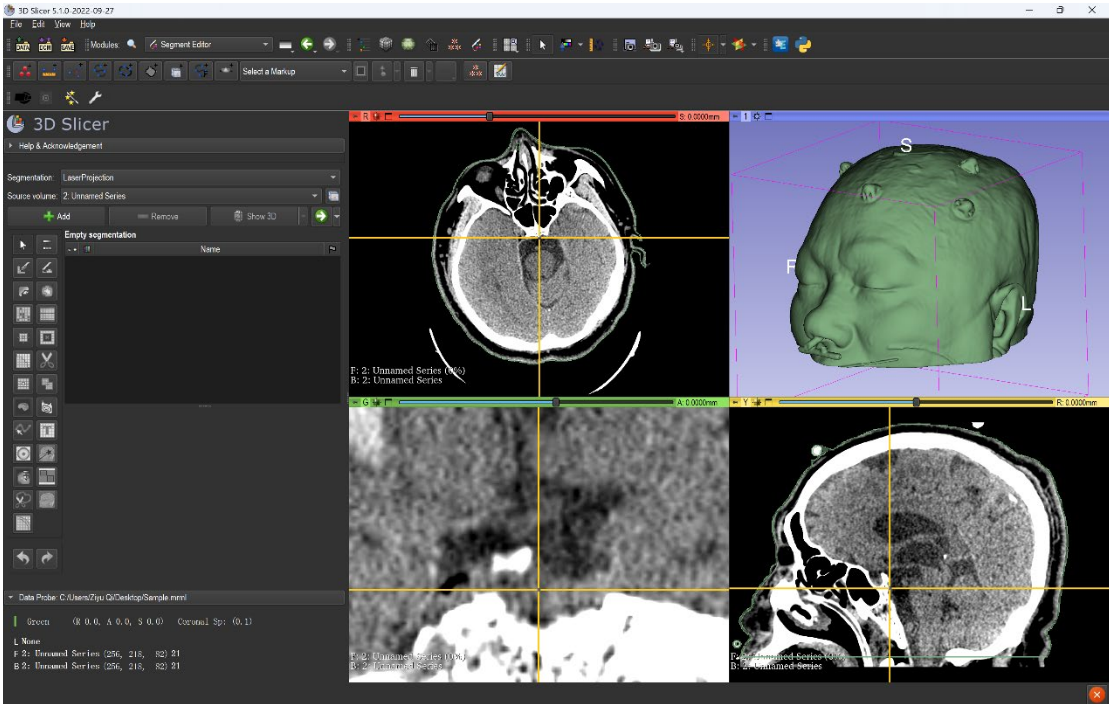

12. Open the Markups module and create three markup point sets (e.g., R/G/Y) to mark the outer contour of the skull in a clockwise direction on each zero plane.

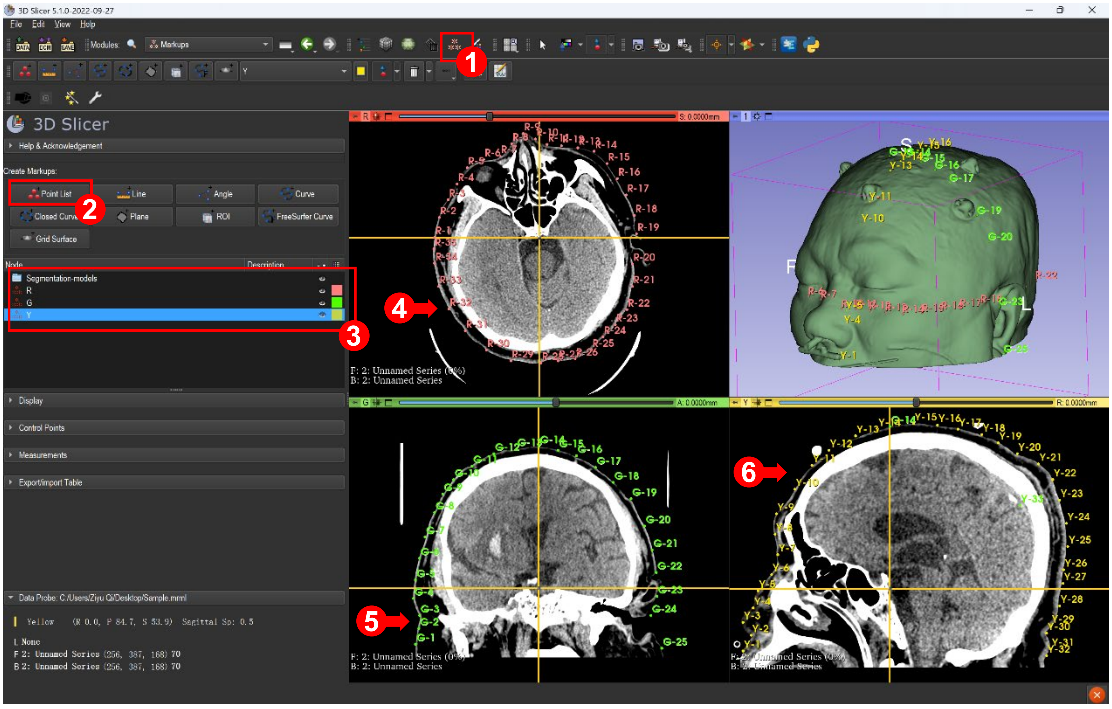

13. Open the CurveMaker module and convert the three point sets into three curve models (disable the Ring mode), representing the laser crosshair projections.

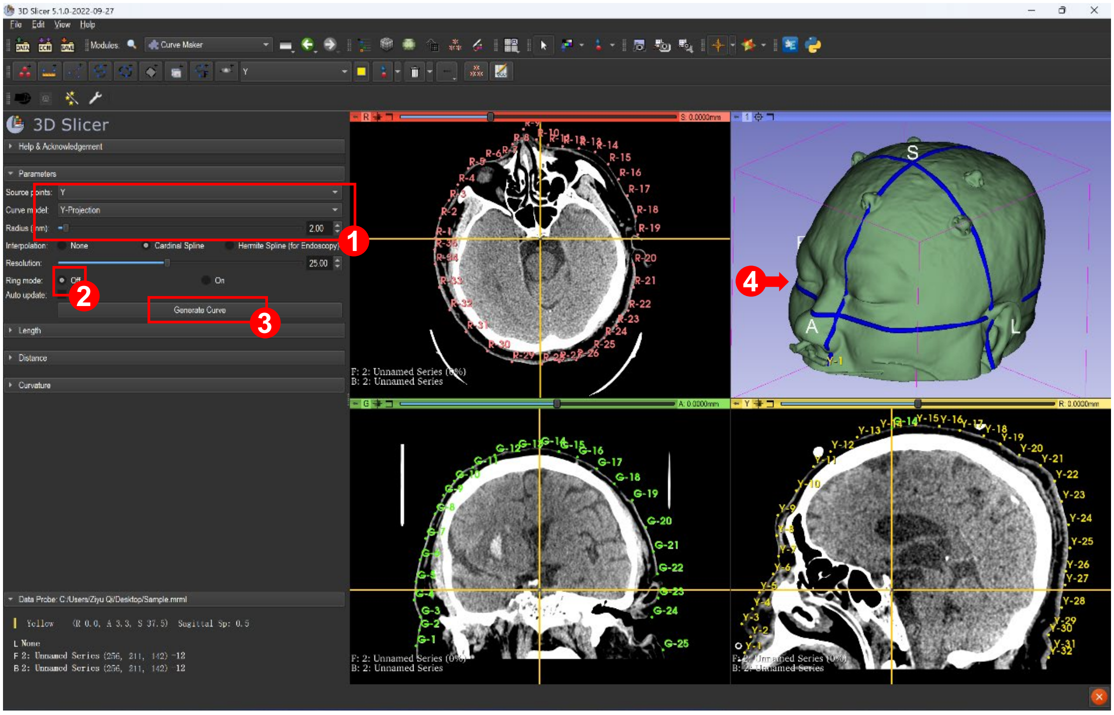

14. Open the MergeModels module and combine the hollow skull model obtained in Step 9 with the three projection lines.

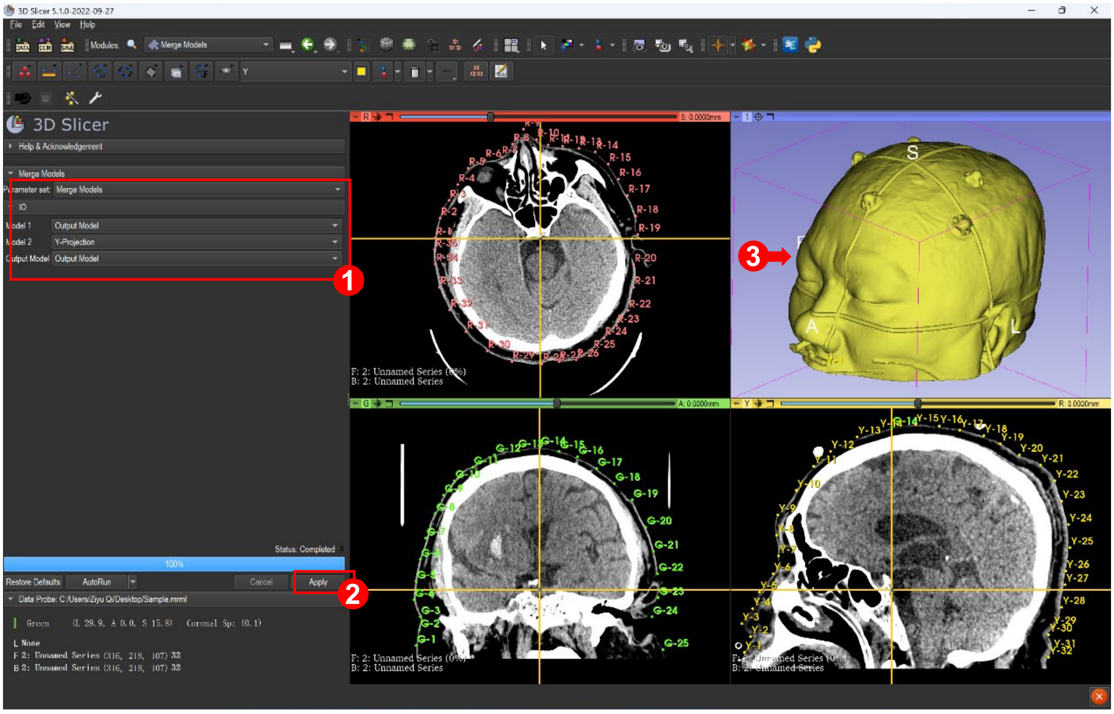

15. Save the final model (OutputModel) in the STL file format.

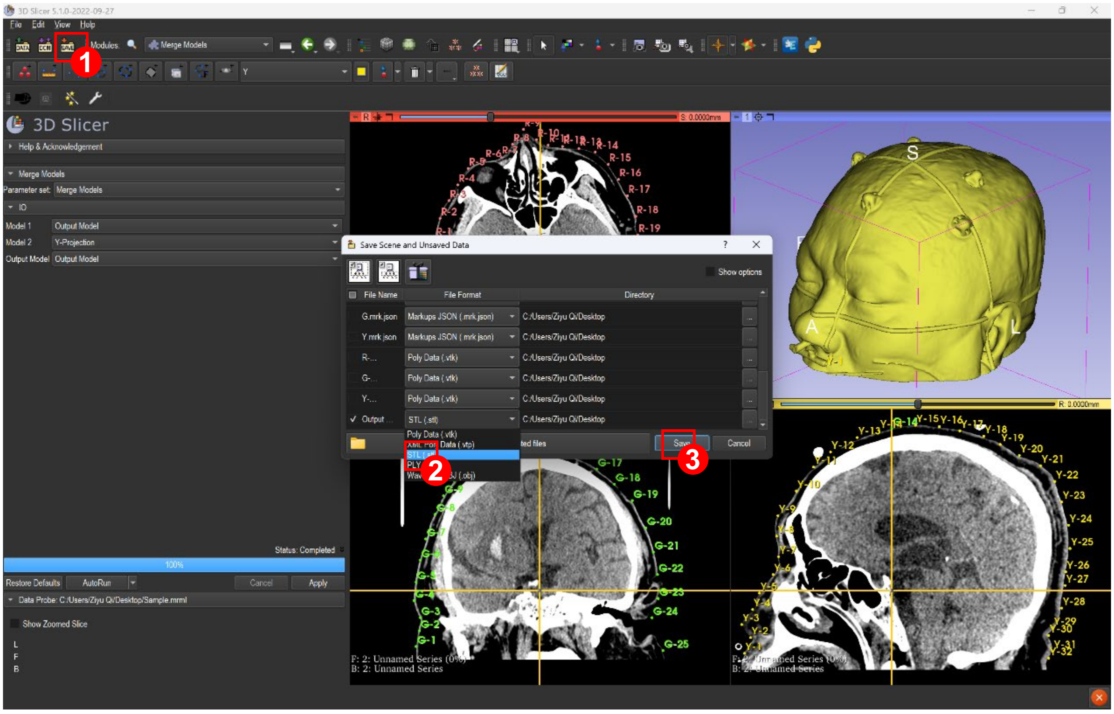

16. Import the STL file into 3D printing slicing software, set the 3D printing parameters, and execute the printing process.

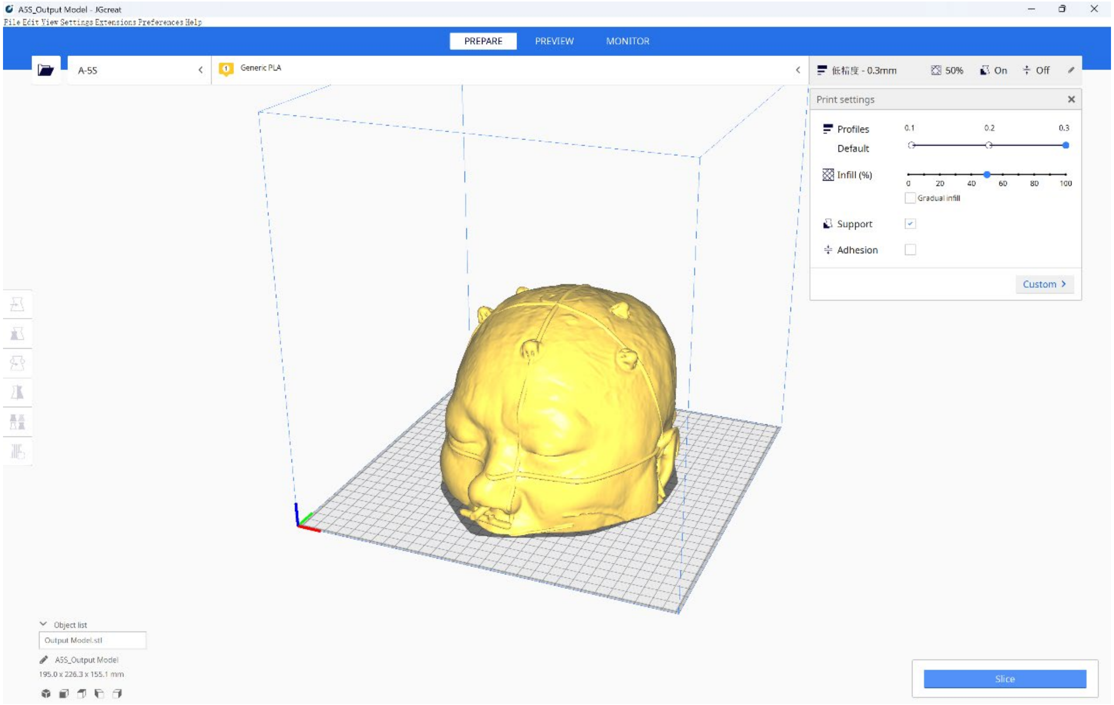

Supplement: Supplementary file 1 [file bioengineering-10-01290-s001.zip › bioengineering-2689696-supplementary/Supplementary Materials/Supplementary Material S1.pdf]
