# Supplementary material for: A Novel Registration Method for a Mixed Reality Navigation System Based on a Laser Crosshair Simulator: A Technical Note
Source: Bioengineering (Basel). 2023 Nov 7;10(11):1290. doi: 10.3390/bioengineering10111290 (PMC10669875; doi:10.3390/bioengineering10111290)
Supplement: Supplementary file 1 [file bioengineering-10-01290-s001.zip › bioengineering-2689696-supplementary/Supplementary Materials/Supplementary Material S2.pdf]

Supplementary Material 2

Protocol: Preparation of Holograms for Validation

Summary

Evaluating the registration accuracy of virtual images or models to real objects requires assessment. In practice, specific anatomical landmarks or artificial markers are often used for quantitative accuracy measurements. However, these specific points lack global averaged characteristics, making the evaluation process less intuitive. This protocol documents a workflow for the qualitative assessment of accuracy using a virtual model. The core idea is to segment the model into eight parts using three orthogonal planes and then retain four non-adjacent sections through an "interval discard" approach. The resulting model includes information about the outer contour and the cutting planes, facilitating the visual evaluation of alignment between the virtual model and the displayed object.

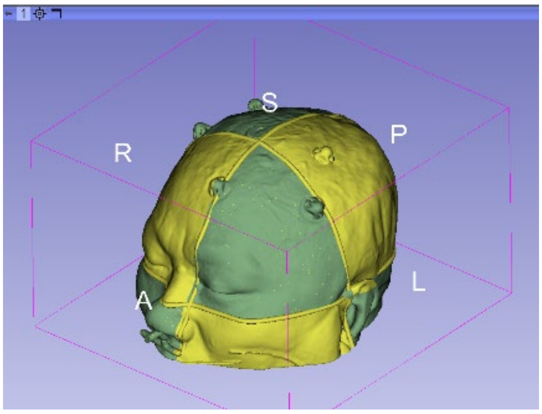

I. Preparation of a Hollow Head Model

1. Follow the steps outlined in the "Protocol: Preparation of a 3D Printed Skull Model with Laser Crosshair Projection Using 3D Slicer Software." Create four copies of the hollow head model and rename them as LAS, RPS, LPI, and RAI.

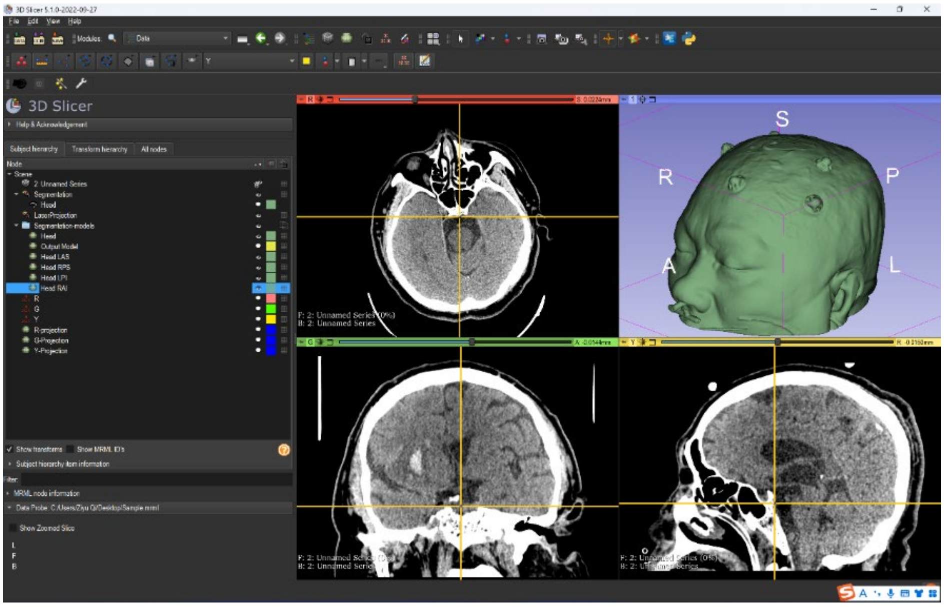

II. Interval Discard of the Model

2. Open the Easy Clip module and adjust the axial, sagittal, and coronal planes individually to the scanning zero plane. Set the clipping parameters for each copy and perform the clipping operation (Clip).

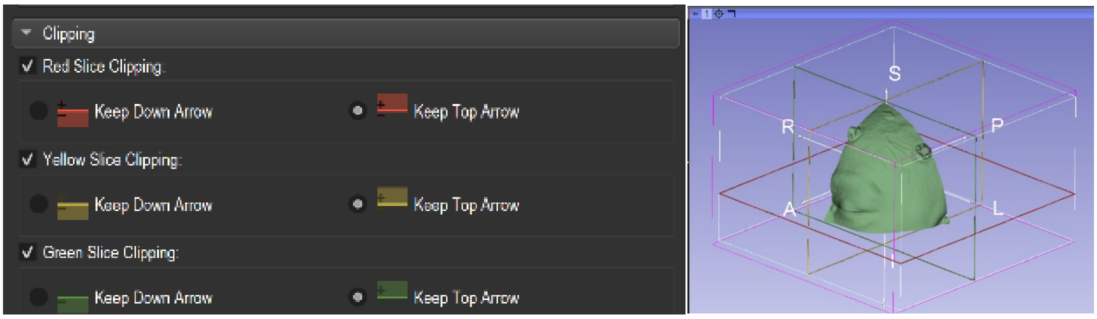

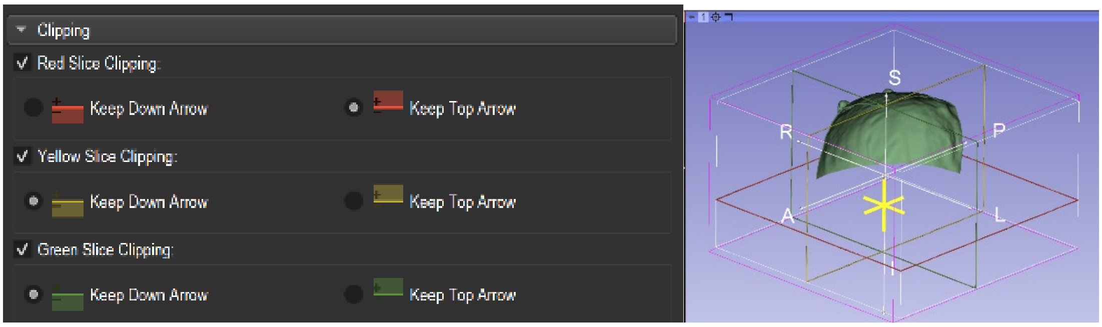

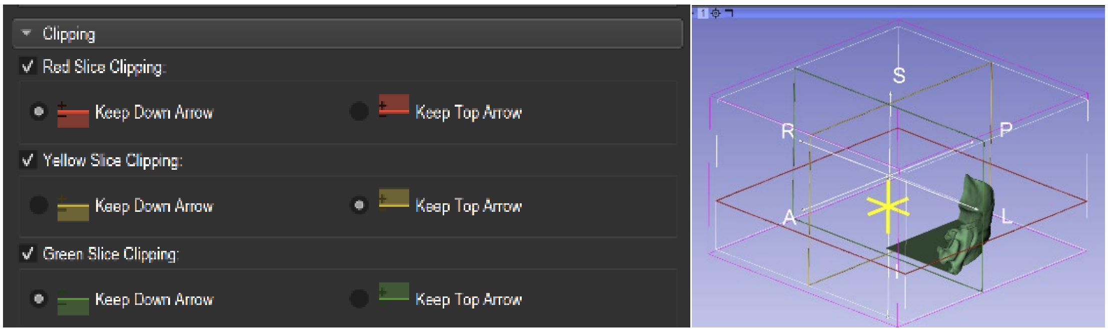

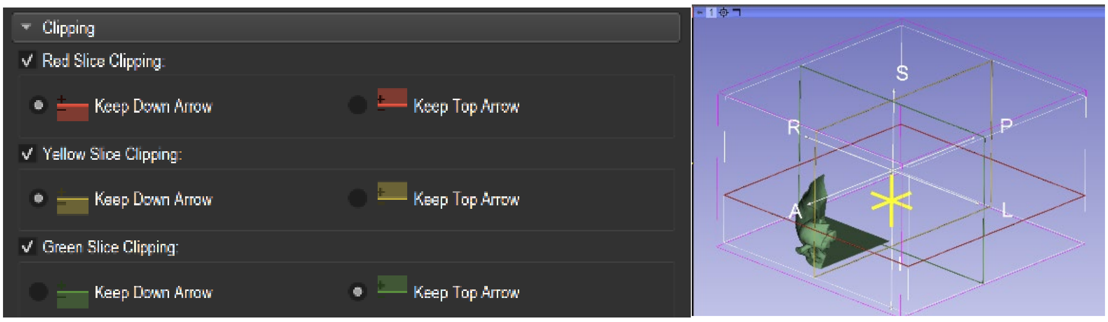

3. Open the MergeModels module and combine the four clipped models into a single model.

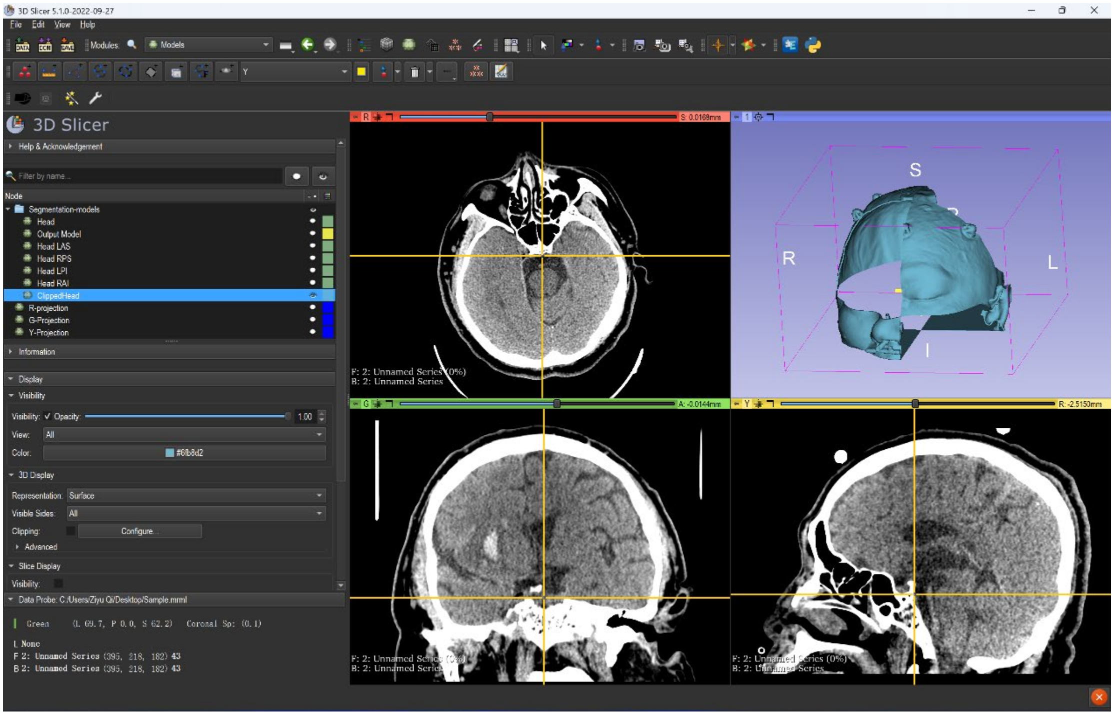

III. Additional Structure Segmentation (Optional)

4. Optionally, use various graphic algorithms in the Segment Editor module for image segmentation or annotation to create corresponding models of hematoma, annotated puncture paths, artificial markers, etc.

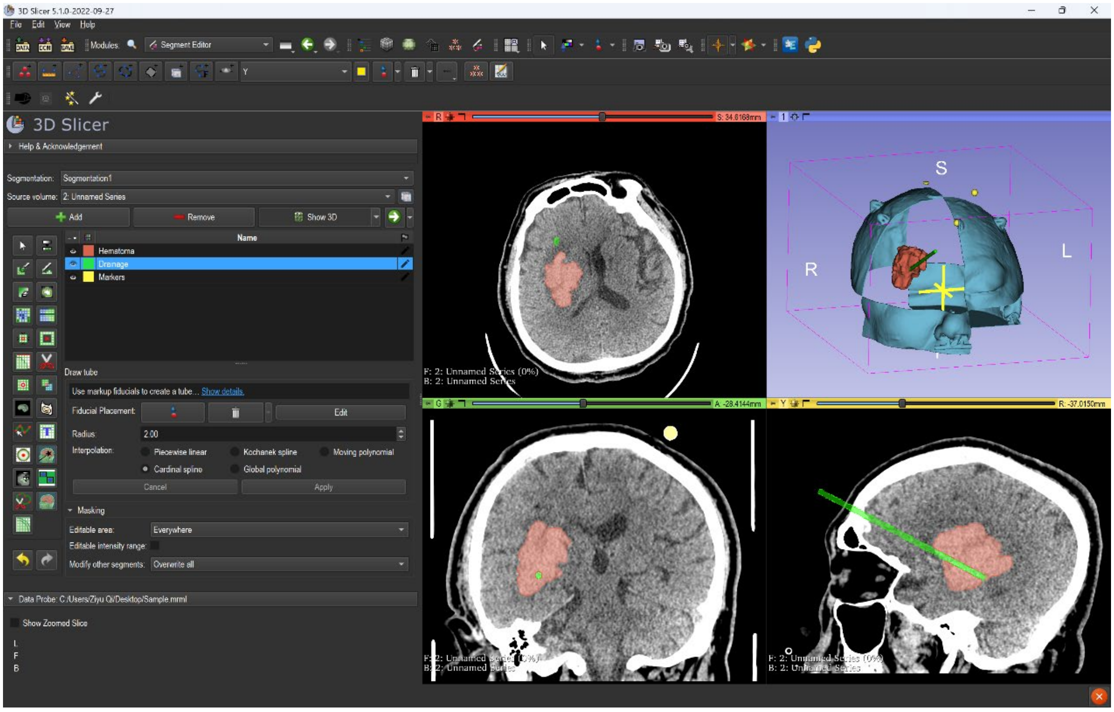

IV. Compatibility Transformation between 3D Slicer and Vuforia SDK (Optional)

5. There is an important difference in the defined direction and scale of coordinate axes between 3D Slicer and Vuforia SDK. Therefore, if further processing of visualized virtual objects using Vuforia SDK is required, a linear transformation must be applied before importing to ensure the correct position, orientation, and size of the objects. The transformation $\boldsymbol{T}_{Slicer}^{Vuforia}$ is known:

$$\boldsymbol{T}_{Slicer}^{Vuforia} = \begin{bmatrix} 0.1 & 0 & 0 & 0 \\ 0 & 0 & -0.1 & 0 \\ 0 & 0.1 & 0 & 0 \\ 0 & 0 & 0 & 1 \end{bmatrix}$$

Geometrically, it involves scaling down the virtual objects in Slicer by a factor of 0.1 and then rotating 90 degrees about the +x-axis in a right-hand manner.

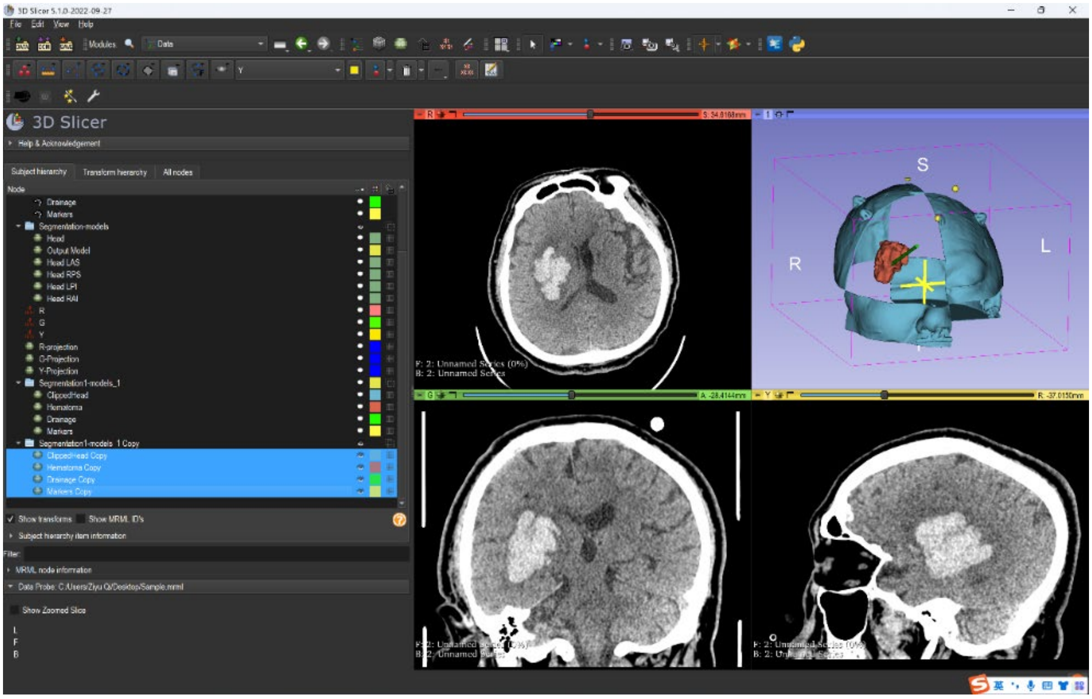

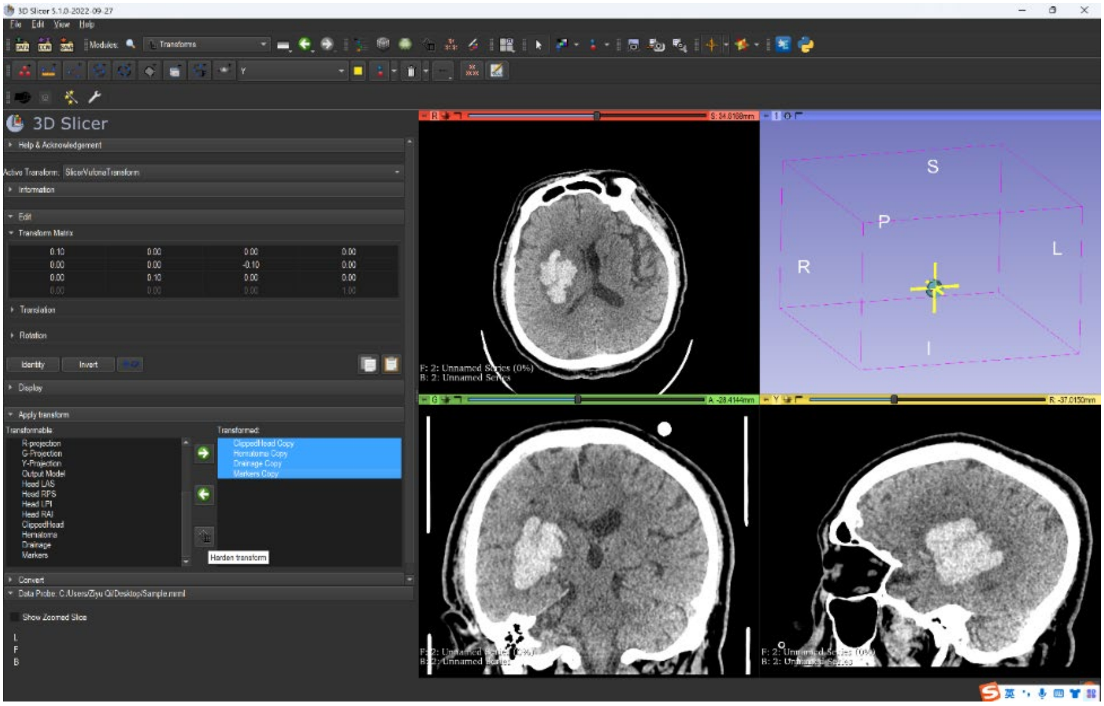
3D Slicer
Help & Acknowledgement
Active Transform: SlicerVuforiaTransform
Information
Edit
Transform Matrix
Translation
Rotation
Identity
Invert
Display
Apply transform
Transformable
Transformed
ClippedHead Copy
Hematoma Copy
Drainage Copy
Markers Copy
Harden transform
Convert
Data Probe: C:/Users/Ziyu Qi/Desktop/Sample.mrml
Show Zoomed Slice
S
P
R
L
I

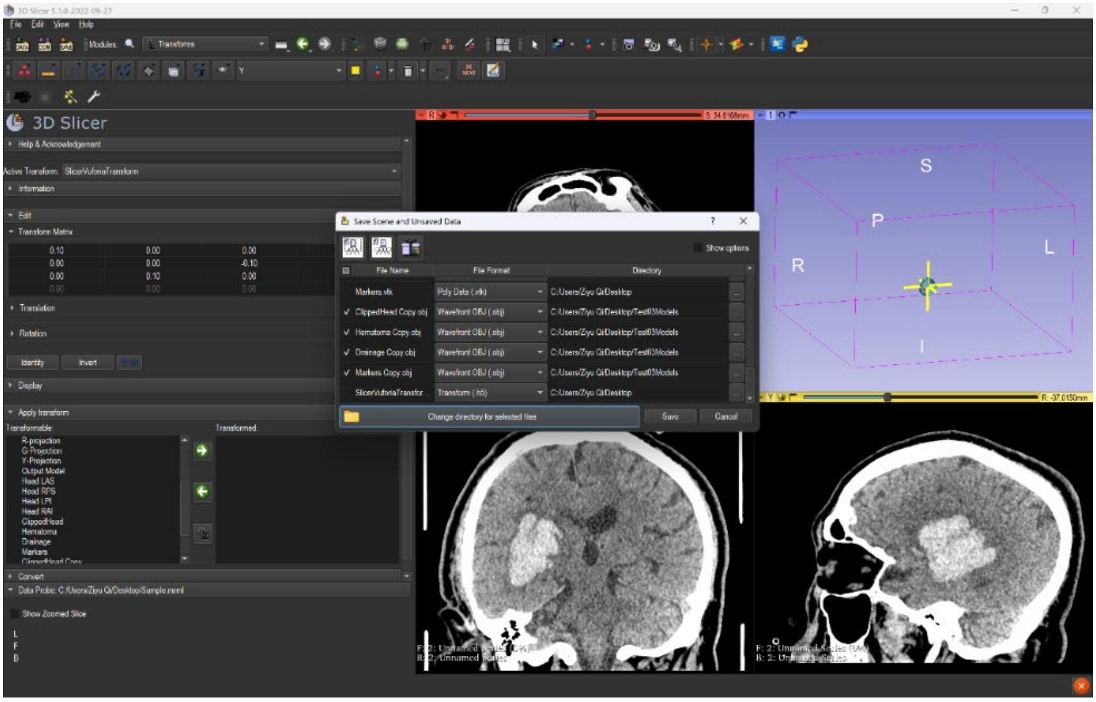
3D Slicer
Save Scene and Unsaved Data
Show options
File Name
File Format
Directory
Markers.vtk
Poly Data (.vtk)
ClippedHead Copy.obj
Wavefront OBJ (.obj)
Hematoma Copy.obj
Drainage Copy.obj
Markers Copy.obj
SlicerVuforiaTransfor...
Transform (.h5)
Change directory for selected files
Save
Cancel
S
P
R
L
I
